# Poly(butylene adipate-co-terephthalate)/Poly(lactic acid) Polymeric Blends Electrospun with TiO_2_-R/Fe_3_O_4_ for Pollutant Photodegradation

**DOI:** 10.3390/polym15030762

**Published:** 2023-02-02

**Authors:** Alessandra Ruyz Medeiros, Fabiana da Silva Lima, Andressa Giombelli Rosenberger, Douglas Cardoso Dragunski, Edvani Curti Muniz, Eduardo Radovanovic, Josiane Caetano

**Affiliations:** 1 Universidade Estadual do Oeste do Paraná—UNIOESTE, Toledo 85903-000, Brazil; 2 Department of Chemistry, State University of Maringá, 5790 Colombo Avenue, Maringá 87020-900, Brazil; 3 Department of Chemistry, Federal University of Piauí, Campus Petronio Portella, Ininga, Teresina 64049-550, Brazil; 4 Department of Material Science, Federal University of Technology—Parana, Estr. dos Pioneiros, 3131, Jardim Morumbi, Londrina 86036-370, Brazil

**Keywords:** polymeric matrix composites, environmental contaminants, electrospinning, semiconductors

## Abstract

This work aimed to use the electrospinning technique to obtain PBAT/PLA polymer fibers, with the semiconductors rutile titanium dioxide (TiO_2_-R) and magnetite iron oxide (Fe_3_O_4_), in order to promote the photocatalytic degradation of environmental contaminants. The parameters used in the electrospinning process to obtain the fibers were distance from the needle to the collecting target of 12 cm, flow of 1 mL h^−1^ and voltage of 14 kV. The best mass ratio of semiconductors in the polymeric fiber was defined from a 2^2^ experimental design, and the values obtained were 10% TiO_2_-R, 1% Fe_3_O_4_ at pH 7.0. Polymer fibers were characterized by Scanning Electron Microscopy (SEM), Differential Scanning Calorimetry (DSC), X-ray Diffraction (XRD), Thermogravimetric Analysis (TGA) and Fourier Transform Infrared (FTIR) techniques. SEM measurements indicated a reduction in fiber diameter after the incorporation of semiconductors; for the PBAT/PLA fiber, the average diameter was 0.9466 ± 0.2490 µm, and for the fiber with TiO_2_-R and Fe_3_O_4_ was 0.6706 ± 0.1447 µm. In the DSC, DRX, TGA and FTIR analyses, it was possible to identify the presence of TiO_2_-R and Fe_3_O_4_ in the fibers, as well as their interactions with polymers, demonstrating changes in the crystallinity and degradation temperature of the material. These fibers were tested against Reactive Red 195 dye, showing an efficiency of 64.0% within 24 h, showing promise for photocatalytic degradation of environmental contaminants.

## 1. Introduction

Currently, one of the great problems that has been pointed out in developed or developing countries is environmental pollution. In recent years, humanity has faced environmental problems, of which the concern with water is extremely relevant. This special attention to water bodies is due to the fact that the demands are growing more and more due to the accelerated increase in population, which generates a greater consumption of quality water. However, the quality of water has decreased alarmingly, and one of the factors that contributes most to this is the incorrect disposal of waste [1].

The absence of standardized procedures that can be applied in the treatment of contaminated water requires that their treatments must be carried out in isolation due to the complexity of pollutants. Thus, the search for new alternatives that allow not only the removal of the pollutant, but the complete mineralization of the contaminants has been studied [2].

Among the commonly used methods are physical-chemical and biological treatments, however, these methods require additional steps at the end of the process, which increases the cost. In view of this, advanced oxidative processes (AOPs) stand out for the possibility of transforming complex compounds into simpler substances with less toxicity through the generation of highly reactive species that can be produced by using strong oxidizing agents, semiconductor metals or radiation [3].

In this context, titanium dioxide (TiO_2_) is a semiconductor that has been extensively studied in AOPs, especially in heterogeneous photocatalysis due to its high chemical stability, low toxicity and low cost. This semiconductor is an insoluble oxide capable of acting in the degradation of microorganisms, such as bacteria and viruses, in addition to organic and inorganic contaminants, as it has the capacity to produce hydroxyl radicals and superoxides from the absorption of ultraviolet light. It has three polymorphic phases, anatase, rutile and brookite. The rutile phase is considered the least photoactive, and one of the possible factors pointing to this is the low oxygen adsorption capacity. However, despite its low photocatalytic activity, rutile has some advantages over the anatase phase, such as the band gap value, which is lower than that of anatase, being 3.0 eV and 3.2 eV, respectively [4,5,6,7].

Despite the advantages, the use of these semiconductors has disadvantages such as the removal of particles from the medium and the recombination of electron-hole charges, mainly from the rutile phase, whose recombination occurs easily and reduces the photocatalytic efficiency. Therefore, the combination of semiconductors with different band gap values has been extensively studied in order to make the photocatalysis process more efficient, making charge recombination difficult [8,9,10].

Thus, the use of iron oxides in photocatalysis has become favorable due to their semiconductor properties, their low cost, high selectivity, high ratio between surface area and volume, possibility of changing the surface and variable band gap of 2.2 to 3.0 eV depending on the oxide used and which facilitates the use of visible light absorption. Iron oxide occurs mainly in the hematite phase (Fe_2_O_3_) and is one of the most used oxides in industrial and scientific fields. However, the magnetite phase (Fe_3_O_4_) is also very promising for photocatalytic studies due to its multiple vacancies. In addition, the magnetic properties can help the separation of the reaction medium and possible reuse of the oxide [11,12,13,14,15,16,17].

However, despite the efficiency of using semiconductors in heterogeneous photocatalysis, some points have become unfavorable, such as the fine suspensions used. These suspensions, as in the case of TiO_2_, require an additional step in the treatment process, as it becomes necessary to separate the photocatalyst to avoid losses. Thus, the immobilization of these photocatalysts has gained relevance due to the ease of separation and reuse capacity [18,19]. Therefore, the use of the electrospinning technique to produce polymeric fibers with large surface area and high porosity containing semiconductors has been highlighted [20].

In the work of Aksimentyeva and collaborators, one can observe the advantage of producing a composite formed by an oxide and a polymer. The authors used nanoparticles of magnetite encapsulated with polymeric casings and modified by barium zirconate and polyaniline and verified, through the characterization of the composite, improvements in the properties of the materials together compared to those separated due to the interactions that reorganize the chemical bonds of the materials. In addition, it is highlighted the fact that the composite formed showed potential for varied applicability such as the development of sensors and materials for the area of medicine [21].

In recent times, polymeric fibers are being developed based on sustainability and the preservation of the environment. Therefore, the use of natural and biodegradable polymers has increasingly attracted the interest of researchers, such as poly(lactic acid) (PLA) and poly(butylene adipate-co-terephthalate) (PBAT), which together form a polymer blend of Ecovio^®^ [22].

Studies show several applications, both for pure polymers and for the PBAT/PLA polymer blend. Rocha [23] incorporated calcium carbonate in Ecovio^®^ fibers to check their behavior and obtained better mechanical properties. Sheikh [24], on the other hand, studied the degradation of Congo Red dye using electrospun fibers of polyvinylpyrrolidone (PVP) incorporated with rutile TiO_2_ and Fe_2_O_3_, and the fibers showed photocatalytic potential degrading about 75% of dye.

Therefore, this study aimed to use the electrospinning process for the development of a PBAT/PLA polymeric fiber incorporated with rutile titanium dioxide and magnetite iron oxide to evaluate its photocatalytic potential in the degradation of the azo dye Reactive Red 195.

## 2. Experimental

### 2.1. Material

The polymer used consists of a polymer blend of 55% poly (butylene adipate-co-terephthalate) (PBAT) and 45% poly (lactic acid) (PLA), manufactured by Basf (Ludwigshafen, Germany), under the name Ecovio^®^ F C2224. This was solubilized in Chloroform (CHCl_3_) and N-N-dimethylformamide (C_3_H_7_NO) available from Neon Comercial (São Paulo, Brazil). The two semiconductors used were obtained from Sigma-Aldrich (St. Louis, MO, USA) with the following characteristics: titanium dioxide-rutile (TiO_2_-R) with 99.9% purity (particle size smaller than 5 µm) and iron oxide-magnetite (Fe_3_O_4_) with 98.0% purity (particle size smaller than 50 nm). The dye used was Reactive Red 195, commercially named Red BF-4B (M_w_ = 1136.31 g mol^−1^; C_31_H_19_N_7_Na_5_O_19_S_6_Cl).

### 2.2. Preparation of Polymer Solution

A 2.5 mL solution was prepared using 15% (*w*/*v*) PBAT/PLA solubilized in a mixture of chloroform and DMF (85:15% *v*/*v*). Subsequently, different percentages of semiconductors were added; for TiO_2_-R, the concentration was varied from 10% to 25% (*w*/*v*), and for Fe_3_O_4_, from 1% to 10% (m/m in relation to the mass of TiO_2_). The solution was subjected to magnetic stirring for 24 h before electrospinning.

### 2.3. Obtaining Polymeric Fibers

Polymeric fibers were obtained from the electrospinning process; for this, the polymeric solution was inserted into a 10 mL glass syringe with a Hamilton needle (Reno, NV, USA) with an internal diameter of 1.5 mm. To control the flow, an SP100I Syringe Pump (Sarasota, FL, USA) was used with an applied flow of 1.0 mL h^−1^. The distance between the needle and the collecting target was 12.0 cm, and the potential difference applied to the high voltage source (Bertan, 30-R, Rio de Janeiro, Brazil) was 14.0 kV. Humidity and temperature were controlled to an average of 45 ± 5% and 25 ± 3 °C, respectively.

### 2.4. Experimental Design

To obtain the polymeric fibers, the mass proportions of the semiconductors were defined using a 2^2^ experimental design with triplicate at the central point. For this, three levels and two factors were considered, as presented in Table 1.

### 2.5. Polymeric Fiber Characterization

To evaluate the morphology of polymeric fibers, samples were subjected to Scanning Electron Microscopy (SEM) (Quanta 250, Waltham, MA, USA). In order to measure the fiber diameter, the software Quantikov Image Analyzer 10.1 (Belo Horizonte, Brazil) was used, and 10 fibers were randomly measured from the SEM images at a magnification of 20 k×.

To identify the structural characteristic of the polymeric fiber and its modifications, it was analyzed by Fourier Transform Infrared (FTIR) spectra, using the Attenuated Total Reflectance (ATR) module. The spectrometer used was a Perkin-Elmer FTIR (Waltham, MA, USA), and the conditions for analysis were a temperature of 25 °C, in the region of 650 to 4000 cm^−1^, with a resolution of 1.0 cm^−1^.

Thermal properties were evaluated by differential scanning calorimetry (DSC). The equipment used was a Shimadzu DSC 60 (Kyoto, Japan), under an inert nitrogen atmosphere with a flow rate of 50.0 mL min^−1^. The heating and cooling rate was 10 °C min^−1^, with heating starting at a temperature of 25 °C to 200 °C, and cooling starting at a temperature of 200 °C to 25 °C. For calculations of the heat involved in the processes and the melting and crystallization temperatures that occurred during heating and cooling, TA60WS software (Kyoto, Japan) was used. The calculation of polymer crystallinity was also performed using Equation (1), conceived by Rossin and Aldhafeeri [25,26].

*X_c_* = (Δ*H_m_* − Δ*H_c_*/Δ*H*°*_m_*) × 100 (1)

where *X_c_* is the degree of crystallinity of the polymer, Δ*H_cc_* is cold crystallization enthalpy of the sample, Δ*H_m_* is fusion enthalpy of the sample, Δ*H°_m_* is theoretical fusion enthalpy of the 100% crystalline sample; in the case of PBAT, Δ*H°_m_* = 114 J·g^−1^, and, for PLA, Δ*H°_m_* = 93.7 J·g^−1^.

Thermal stability of the fibers was verified by thermogravimetric analysis (TGA) with Perkin-Elmer STA 6000 equipment (Waltham, MA, USA). The experimental conditions were a nitrogen atmosphere with a flow rate of 50 mL min^−1^ at a heating rate of 10 °C min^−1^ and temperature varied from 25 to 545 °C.

X-ray diffraction analysis was performed to verify the crystallinity of the samples. For this, a Bruker^®^ diffractometer (Billerica, MA, USA) was used, with diffraction at a 2θ angle, ranging from 7° to 80°, with an increment of 0.01°, using Cu_Kα_ radiation of λ = 1.5406 Å, as a graphite monochromator.

### 2.6. Photocatalytic Degradation

To assess the photocatalytic capacity of the fibers obtained, an aqueous solution was prepared, at a concentration of 35 mg L^−1^, of the azo dye Reactive Red 195, and 30 mL of solution was placed in a beaker containing the polymeric fiber in a steel support of 3.0 cm^−1^ in diameter. Subsequently, the dye solution was irradiated by an Osram Ultra-Vitalux 300 W UV-A/UV-B Xenon lamp (Munich, Germany) with a measured intensity of 46 W m^−2^ for a period of 1440 min. The distance between the lamp and the solution was measured at 28.5 cm, and a thermostated bath kept the temperature of the study system controlled at 21.0 °C.

Dye solution degradation was evaluated using a Shimadzu-1800 UV/VIS spectrophotometer (Kyoto, Japan) in the 200–800 nm spectral region. For this analysis, aliquots were taken at defined times up to 1440 min. All aliquots taken for analysis were returned to the irradiated solution.

## 3. Results and Discussion

### 3.1. Preliminary Tests

**Figure 1:**
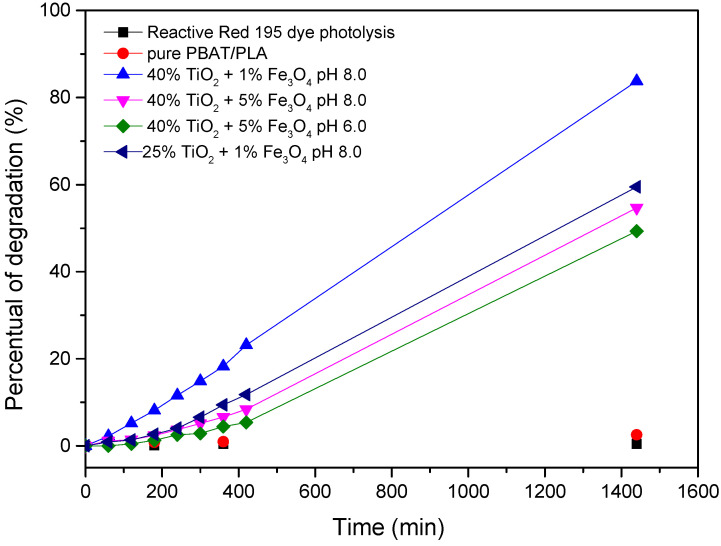
Degradation of BF-4B Reactive Red dye (35 mg L^−1^) for polymeric fibers of pure PBAT/PLA; incorporated and the photolysis of the dye at pH 7 under luminous intensity of 46 W m^2^.

The mass proportions of semiconductors (TiO_2_-R/Fe_3_O_4_) with higher photocatalytic efficiency were defined using a 2^2^ factorial design. For this study, preliminary degradation tests were performed using the reactive dye Reactive Red BF-4B in order to obtain the values to be applied in the design. A small variation in the pH of the medium was also produced to check the influence on the analyte degradation. Figure 1 illustrates the preliminary tests, together with the dye photolysis and the study with the polymeric fiber with the PBAT/PLA polymer alone.

Within 24 h of exposure to light, there was no dye degradation and the pure PBAT/PLA fiber also did not show photocatalytic activity in dye degradation. A lower percentage of Fe_3_O_4_ caused an increase in degradation of approximately 30% for the 40% TiO_2_-R + 1% Fe_3_O_4_ fiber. According to the catalytic regime, the degradation speed is generally proportional to the catalyst mass, but there is a limit on the amount of mass to be used, and, after reaching it, the process becomes independent of it. This maximum amount depends on some factors, such as the experimental conditions, light irradiation and also the amount of dopant, which, if excessive (above 1%), causes a greater number of defects in the doped structure, and, thereby, the degradation response decreases [27,28].

Furthermore, there was little difference in dye degradation when comparing the fiber containing 40% TiO_2_-R + 5% Fe_3_O_4_ at pH 6.0 and 8.0. Due to the small difference between the pH tested, it was decided to carry out the experimental design at pH 7.0, as more acidic or alkaline pH values can favor the hydrolysis of polymer fibers; in addition, in acidic pH, iron solubilization can occur.

After these tests, fiber containing 40% TiO_2_ was visibly dry and fragile, therefore, we decided to do a test with 25% TiO_2_ mass, setting the Fe_3_O_4_ mass at 1%. The degradation obtained was almost 60% of dye and, visually, the fiber produced was more resistant.

From the preliminary tests, it was possible to set the levels of TiO_2_ and Fe_3_O_4_ applied in the 2^2^ design. The experiments were performed randomly and irradiated for 24 h; the evaluated response was the percentage of degradation of the dye Reactive Red 195. The results are listed in Table 2.

Only experiment 1 presented a degradation percentage higher than 50%. Thus, to assess which factor has the greatest influence on the response variable, Table 3 was analyzed.

**Figure 2:**
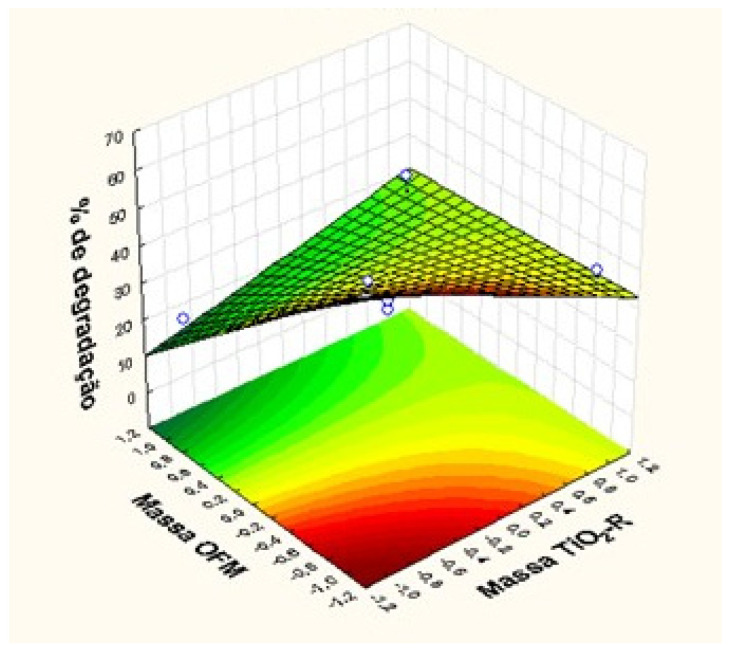
Surface graphics for TiO_2_-R/Fe_3_O_4_ ratio.

Table 3 shows that the interaction effects related to the TiO_2_/Fe_3_O_4_ ratio, as well as the Fe_3_O_4_ mass, are significant, noting that iron oxide is an important factor for dye degradation. These results can be interpreted according to the algebraic signs of the effects. For the main effects with negative values, there is an indication that the response increases when the factor moves towards its minimum level. For interactions, positive values show that the two factors must go towards the same level, being this lower or higher, for the response to increase. Therefore, the surface graph was constructed, as shown in Figure 2.

It can be seen in Figure 2 that the increase in degradation occurs with the decrease in the percentage of Fe_3_O_4_ and TiO_2_-R mass, which corroborates the studies shown in Table 2, with the polymeric fiber with TiO_2_/Fe_3_O_4_ at 10:1% with better photocatalytic activity.

### 3.2. Polymeric Fiber Characterization

**Figure 3:**
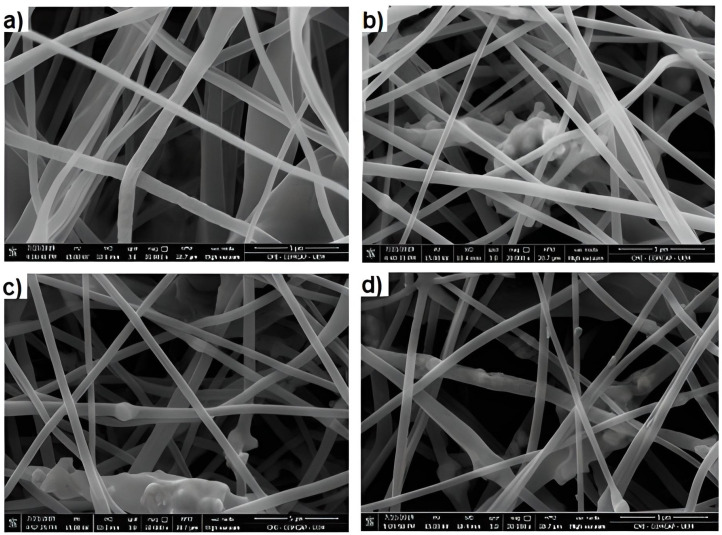
Scanning Electron Microscopy (SEM) images of (**a**) pure PBAT/PLA polymeric fibers; (**b**) with 10% TiO_2_-R + 1% Fe_3_O_4_; (**c**) with 10% TiO_2_-R; and (**d**) with 1% Fe_3_O_4_. All at 20,000× magnification.

After obtaining the polymeric fibers, SEM analyses were performed (Figure 3) in order to assess diameters, morphologies and their possible alterations.

**Figure 4:**
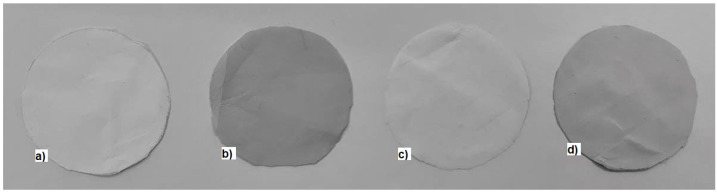
Images of polymeric fibers of PBAT/PLA (**a**) with 10% TiO_2_-R; (**b**) with 1% Fe_3_O_4_; (**c**) pure; and (**d**) with 10% TiO_2_-R + 1% Fe_3_O_4_.

Analyzing the images, it can be seen that both the fibers with embedded semiconductors and the pure PBAT/PLA fiber presented a uniform and continuous appearance. Moreover, it is noted that there is evidence that TiO_2_-R and Fe_3_O_4_ are found inside the fibers. In addition, in the presence of semiconductors, there was a reduction in their diameter, and, for the PBAT/PLA fiber, the mean diameter was 0.9466 ± 0.2490 µm, for TiO_2_-R, 0.5890 ± 0.1707 µm, for Fe_3_O_4_, 0.7102 ± 0.2479 µm and for TiO_2_-R with Fe_3_O_4_, 0.6706 ± 0.1447 µm. When analyzing the standard deviation of the pure PBAT/PLA fiber (±0.2490 µm) and the fiber incorporated with the two semiconductors (±0.1447 µm), it was observed that there was an increase in homogeneity with their presence. This occurs because the presence of semiconductors possibly increases the electrical conductivity of the polymer solution [29]. In addition to SEM images, photographs were taken (Figure 4) of polymer fibers with and without TiO_2_-R and Fe_3_O_4_.

**Figure 5:**
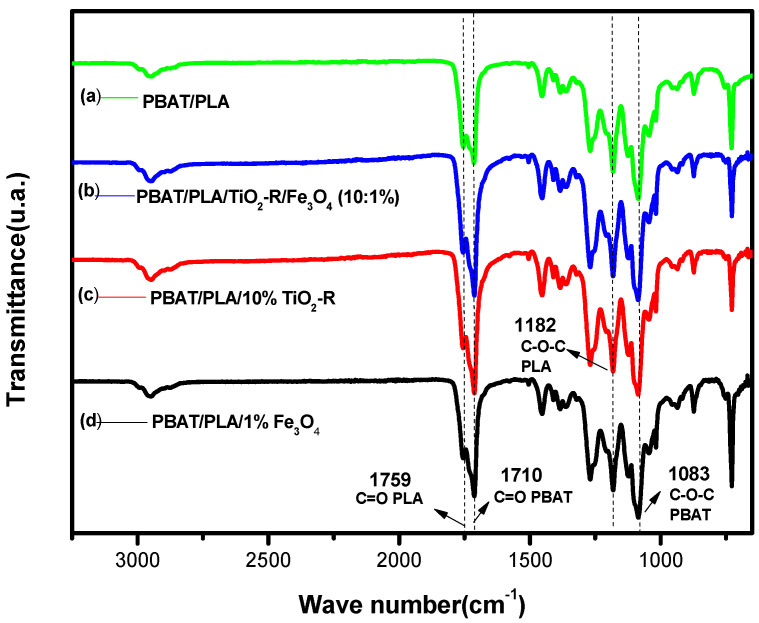
Fourier Transform Infrared (FTIR) vibrational spectra from attenuated reflectance (ATR) for PBAT/PLA polymer fibers.

From the images, fibers 4-a and 4-c showed little difference in color, both of which have a white color due to the characteristics of the materials. However, in the 4-a image, the white color is more intense due to the presence of TiO_2_-R. Fibers 4-b and 4-d, on the other hand, exhibited a gray coloration, whereas fiber 4-b had a darker color because it contains only Fe_3_O_4_. Given this and the SEM images, it can be said that the fibers are possibly found in the presence of semiconductors [30,31]. To prove the semiconductors in polymer fibers, FTIR analyses were performed, as illustrated in Figure 5.

A summary of the peaks observed in the FTIR analysis are presented in Table 4.

From the infrared spectra, it was possible to observe the characteristic bands of PBAT and PLA polymers, the bands being presented for the ester group for PBAT at 1083 cm^−1^, and for PLA at 1182 cm^−1^. Furthermore, another characteristic band is the carbonyl of polymers, which is presented for PBAT and PLA at 1710 cm^−1^ and 1759 cm^−1^, respectively. The band observed at 2950 cm^−1^ refers to the stretching of C–H bonds [32]. Regarding the incorporation of semiconductors, no characteristic bands were observed. However, there was an increase in the intensity of the carbonyl band of both polymers, especially the C=O band of PBAT, when compared to the pure polymer fiber, which may show that the semiconductors are found inside the fibers.

**Figure 6:**
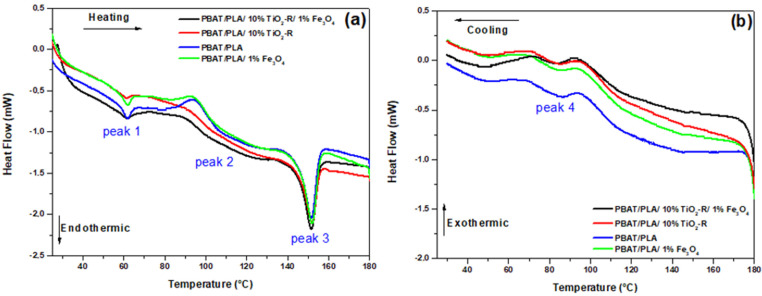
(**a**) Heating and (**b**) cooling thermograms for PBAT/PLA polymeric fibers.

In order to assess the thermal stability of the fiber containing the semiconductors, DSC analyses were performed (Figure 6).

According to thermograms, it is possible to identify three endothermic peaks (Figure 6a), referring to the PBAT/PLA blend; these being peak 1 (≈62.0 °C), peak 2 (≈90.0 °C) and peak 3 (≈150.0°). Peak 1 is associated with the glass transition temperature (T_g_) of PLA, that is, the passage from a rigid material to a more organized material. Peak 2 refers to the melting temperature (T_f_) of PBAT, and peak 3 to T_f_ of PLA [33,34].

T_f_ of PBAT has a broader and less intense peak compared to T_f_ of PLA, which has a more intense behavior. This may indicate that PBAT has less crystallinity, that is, it has less rigidity. Studies report that PBAT has two melting temperatures, one referring to the melting of the aliphatic part (close to the T_g_ of PLA (peak 1)), and the other temperature referring to the aromatic part (peak 2). Therefore, due to a possible interaction between PBAT/PLA, the T_f_ of PBAT, referring to the aliphatic part, may have been overlaid by the T_g_ of PLA [35,36].

In addition to the endothermic peaks, it was possible to observe in Figure 6b the presence of one exothermic peak for polymer fibers at approximately 95 °C. This peak is related to the crystallization temperature (T_cc_) of PLA [35]. The melting and crystallization temperatures and the heat involved in the processes are described in Table 5.

With the presence of semiconductors, both for the fibers containing the blend and for those containing the semiconductors separately, there was a decrease in the melting and crystallization temperatures of PBAT and PLA, but they were not very expressive. The heat involved in the melting process, on the other hand, decreased more sharply, which may indicate the interaction of TiO_2_ and Fe_3_O_4_ with both polymers. To better assess this possible interaction, the degree of crystallinity related to the fusion process of PBAT and PLA was calculated. The results are listed in Table 6.

After the incorporation of semiconductors, a decrease in the crystallinity of both PBAT and PLA was found, which may indicate that semiconductors interacted with both polymers. The polymeric fiber containing only TiO_2_ has a lower crystallinity value, that is, the incorporation of this semiconductor makes the polymeric fibers less organized, which may indicate that it worked as a plasticizer. In addition, it is noted that, for PBAT, there was a reduction in crystallinity for both polymer fibers; this may be due to PBAT having aromatic chains and possibly they fold TiO_2_, unlike PLA, which has aliphatic chains and can therefore form clusters with the semiconductor [37].

**Figure 7:**
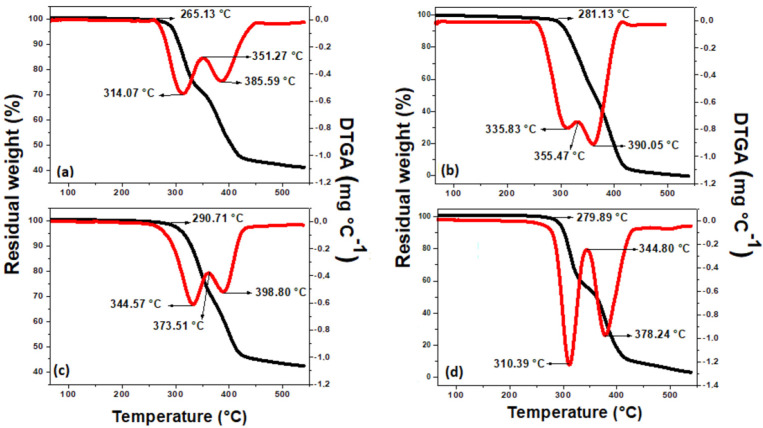
Thermogravimetric curves (TGA) and derivative of degradation curves (DTGA) of pure (**a**) PBAT/PLA polymeric fibers; (**b**) with 10% TiO_2_—R; (**c**) with 1% Fe_3_O_4_; and (**d**) with 10% TiO_2_—R + 1% Fe_3_O_4_.

To verify whether the degradation temperature of the PBAT/PLA fiber was changed in the presence of semiconductors, TGA measurements were performed, as can be seen in Figure 7.

Both polymer fibers lost mass in two thermal events defined by derivative curves, in which the first thermal event indicates the degradation of the PLA polymer and the second, the PBAT polymer [38].

Furthermore, it was possible to verify that the presence of TiO_2_ and Fe_3_O_4_ modified the degradation temperature of polymeric fibers in relation to pure PBAT/PLA. For fibers containing only 10% TiO_2_—R, there was an increase in the initial degradation temperature for both polymers, being ≈9.0 °C for PLA and ≈20 °C for PBAT, showing an increase in their thermal stability.

However, for polymer fibers containing Fe_3_O_4_, both pure and in the presence of TiO_2_, there was a decrease in the initial degradation temperatures. For pure Fe_3_O_4_ fiber, this decrease was ≈2.0 °C for PLA, and ≈10.0 °C for PBAT, indicating a more pronounced decrease in stability for PBAT. In addition, a change in the DTGA profile in the presence of semiconductors is noted, indicating that Fe_3_O_4_ possibly interacts with polymers.

As for the polymeric fiber containing TiO_2_ and Fe_3_O_4_, this decrease in the initial degradation temperature was ≈15.0 °C for PLA, and ≈4.0 °C for PBAT, indicating a lower stability for PLA. Therefore, these results can show that there is a more synergistic effect for PLA when the two semiconductors are combined, showing that, even in small amounts, iron can influence the characteristics of the polymeric fiber, corroborating the degradation tests. Furthermore, these results may indicate interaction between semiconductors and polymers, as also shown by DSC analysis.

**Figure 8:**
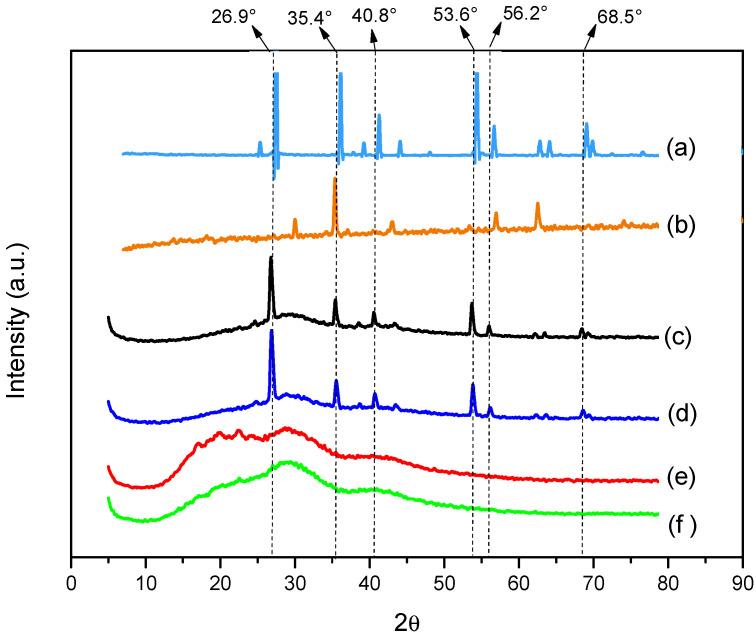
X-ray diffractograms of powder samples of (**a**) TiO—R and (**b**) Fe_3_O_4_ and polymeric fibers of PBAT/PLA (**c**) with 10% TiO_2_—R + 1% Fe_3_O_4_; (**d**) with 10% TiO_2_—R; (**e**) with 1% Fe_3_O_4_; and (**f**) fiber in the absence of semiconductor.

XRD analyses (Figure 8) were performed to assess fiber crystallinity before and after addition of semiconductors.

In Figure 8, a change in the characteristics of the polymeric fiber can be seen after the incorporation of semiconductors [39]. Between the angles of 25.0° and 70.0°, there was an increase in crystallinity, which is probably due to the crystal structure of semiconductors, as can be seen in the diffractograms (Figure 8a,b) of the powder samples. However, when comparing polymer fibers in the presence of semiconductors separately (Figure 8d,e), it can be noted that the crystallinity of TiO_2_-R is more evident than Fe_3_O_4_, corroborating Figure 8c, which shows crystallinity peaks similar to those of powdered TiO_2_, indicating that rutile titanium dioxide is responsible for the increase in crystallinity of the fiber containing the semiconductor blend. These results, together with the SEM, TGA, DSC and FTIR analyses, indicate the presence of semiconductors in polymer fibers.

### 3.3. Photocatalytic Degradation of Reactive Red 195

**Figure 9:**
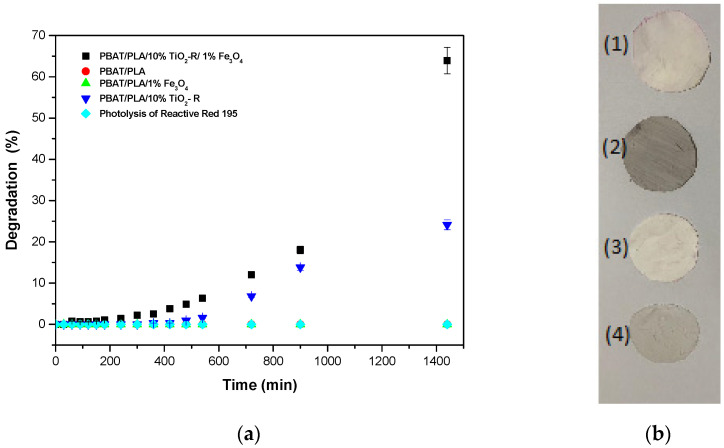
(**a**) Degradation of Reactive Red 195 for pure PBAT/PLA fibers and incorporated PBAT/PLA fibers. (**b**) Images of membrane nanostructure of PBAT/PLA after 1400 min of photodegradation ((1) with 10% TiO_2_—R; (2) with 1% Fe_3_O_4_; (3) pure; and (4) with 10% TiO_2_—R + 1% Fe_3_O_4_).

The potential of the polymeric fiber was tested by photocatalytic degradation, using the dye Reactive Red 195 as a model molecule. The tests were performed with the polymeric fibers in the absence of semiconductors (photolysis), in the presence of each one separately and in the fibers containing the blend, as shown in Figure 9.

From these tests, it is observed in Figure 9a that there was no dye degradation with light irradiation, and that the pure PBAT/PLA polymeric fiber and that with 1% Fe_3_O_4_ did not present photocatalytic activity.

Furthermore, the polymeric fiber containing the blend of TiO_2_—R/Fe_3_O_4_ semiconductors had greater photocatalytic activity (64.0%), compared to polymeric fibers containing only TiO_2_ (24.0%), for a degradation time of 1440 min. According to Zhan [40], the improvement in the photocatalytic efficiency of the polymeric fiber of titanium dioxide with iron oxide may occur because the iron oxide possibly increases the titanium dioxide absorption intensity in the visible light region and, consequently, more electrons and holes are generated and accelerate photocatalysis, in addition to decreasing the electron-hole recombination time.

Furthermore, in Figure 9b, it can be seen that, after a degradation time of 1440 min, all membranes used in the test were visually stable, which may indicate that they can be reused.

**Figure 10:**
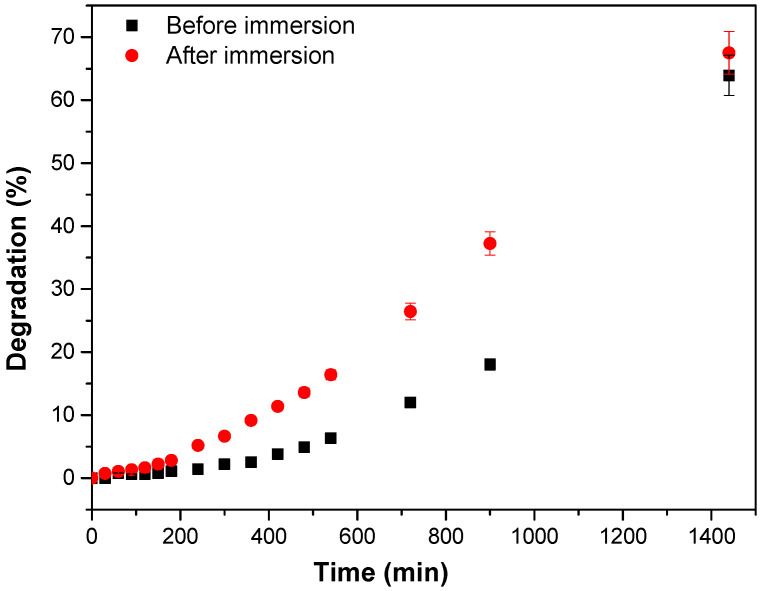
Wettability of PBAT/PLA fibers incorporated with TiO_2_-R/Fe_3_O_4_ (10:1%) in the degradation of the dye Reactive Red 195.

Another important factor is the onset of degradation, which only occurs after 300 min. This is possibly due to the hydrophobic character of the PBAT/PLA blend, making it difficult for semiconductors to contact the dye [41]. Therefore, it was decided to test the degradation with the polymeric fiber, after its immersion for 1080 min in distilled water, as illustrated in Figure 10.

The polymeric fiber immersed in water presented better photocatalytic efficiency compared to the fiber without immersion, being about 20.0% higher after 900 min. These results indicate that, with immersion in water, fibers may swell, causing the dye to come into contact with the semiconductors, promoting more rapid degradation.

These tests showed that fibers containing the semiconductor have the potential to promote photocatalytic degradation of environmental contaminants.

## 4. Conclusions

It was possible to obtain a polymeric fiber of PBAT/PLA/TiO_2_-R/Fe_3_O_4_ with photocatalytic activity by the electrospinning process, using experimental design. The characterization of the fibers showed the presence of semiconductors with a decrease in the average diameter and an increase in uniformity, indicating possible interactions. These results demonstrated that semiconductors can be immobilized in polymeric fibers obtained by electrospinning without impairing their properties and photocatalytic characteristics. Moreover, that, because of this, the material produced has an interesting applicability in the degradation of pollutants with the possibility of reuse without considerable loss of its efficiency for more than one cycle of degradation, in addition to facilitating the separation of semiconductors from the reaction medium once they remain immobilized in the polymeric fiber.

## Figures and Tables

**Table 1:** Experimental design.

Factors	(−1)	(0)	(+1)
**TiO_2_-R (% *w*/*w*)**	10.0	17.5	25.0
**Fe_3_O_4_ (% *w*/*w*)**	1.0	5.5	10.0

**Table 2:** Matrix of the 2^2^ experimental design.

Order of Experiments	TiO_2_-R Mass (%)	Fe_3_O_4_ Mass (%)	Dye Degradation Percentage (%)
3	10.0	10.0	20.7
5	17.5	5.5	28.7
6	17.5	5.5	32.3
2	25.0	1.0	39.6
1	10.0	1.0	63.9
7	17.5	5.5	26.1
4	25.0	10.0	33.9

**Table 3:** Effects for dye degradation percentage response.

	Effects	Standard Error	*t*-Value	*p* *-Value
Intercept	**35.028**	**1.176**	**29.766**	**0.001**
(1) TiO_2_-R mass	−5.550	3.113	−1.782	0.216
(2) Fe_3_O_4_ mass	**−24.450**	**3.113**	**−7.853**	**0.015**
1 by 2	**18.750**	**3.113**	**6.022**	**0.026**

* Values in red show the significant variables at the 95% confidence interval (*p*-value < 0.05%).

**Table 4:** Absorption band in the infrared region for PBAT and PLA.

Polymer	Wave Number (cm^−1^)	Functional Group
PLA	1759	C=O
PBAT	2948 and 2886	C–H sp³ and sp²
PBAT	1710	C=O
PLA	1453 and 1392	C–H sp²
PLA	1182	C–O–C
PBAT	1083	C–O–C

**Table 5:** Temperatures and heat involved in the fusion and crystallization of polymer fibers.

Polymer Fiber	Peak 1		Peak 2		Peak 3		Peak 4	
	T_fusion_ (°C)	Heat (J·g^−1^)	T_fusion_ (°C)	Heat (J·g^−1^)	T_fusion_ (°C)	Heat (J·g^−1^)	T_crystallization_ (°C)	Heat (J·g^−1^)
PBAT/PLA	61.92	1.46	94.95	11.07	151.71	11.77	94.07	−0.08
PBAT/PLA/10% TiO_2_-R	61.64	0.76	87.93	5.48	151.63	7.43	93.41	−0.09
PBAT/PLA/1% Fe_3_O_4_	61.84	1.00	94.01	9.74	151.51	11.23	93.04	−0.09
PBAT/PLA/10% TiO_2_-R/1% Fe_3_O_4_	61.86	0.90	90.45	5.90	151.54	10.66	92.67	−0.09

**Table 6:** Crystallinity percentages calculated for the melting peaks of the polymeric fibers.

Polymer Fiber	Crystallinity Peak 2 (%)	Crystallinity Peak 3 (%)
PBAT/PLA	9.78	10.40
PBAT/PLA/10% TiO_2_-R	5.15	6.60
PBAT/PLA/1% Fe_3_O_4_	8.62	9.92
PBAT/PLA/10% TiO_2_-R/1% Fe_3_O_4_	5.25	9.42

## Data Availability

The data presented in this study are available in the article.
